# Inferring bacteriophage infection strategies from genome sequence: analysis of bacteriophage 7-11 and related phages

**DOI:** 10.1186/1471-2148-15-S1-S1

**Published:** 2015-02-02

**Authors:** Jelena Guzina, Marko Djordjevic

**Affiliations:** 1 Faculty of Biology, University of Belgrade, Studentski trg 16, 11000 Belgrade, Serbia

**Keywords:** bacteriophages, genome analysis, promoter specificity, RNA polymerase, transcription regulation

## Abstract

**Background:**

Analyzing regulation of bacteriophage gene expression historically lead to establishing major paradigms of molecular biology, and may provide important medical applications in the future. Temporal regulation of bacteriophage transcription is commonly analyzed through a labor-intensive combination of biochemical and bioinformatic approaches and macroarray measurements. We here investigate to what extent one can understand gene expression strategies of lytic phages, by directly analyzing their genomes through bioinformatic methods. We address this question on a recently sequenced lytic bacteriophage 7 - 11 that infects bacterium *Salmonella enterica*.

**Results:**

We identify novel promoters for the bacteriophage-encoded σ factor, and test the predictions through homology with another bacteriophage (phiEco32) that has been experimentally characterized in detail. Interestingly, standard approach based on multiple local sequence alignment (MLSA) fails to correctly identify the promoters, but a simpler procedure that is based on pairwise alignment of intergenic regions identifies the desired motifs; we argue that such search strategy is more effective for promoters of bacteriophage-encoded σ factors that are typically well conserved but appear in low copy numbers, which we also verify on two additional bacteriophage genomes. Identifying promoters for bacteriophage encoded σ factors together with a more straightforward identification of promoters for bacterial encoded σ factor, allows clustering the genes in putative early, middle and late class, and consequently predicting the temporal regulation of bacteriophage gene expression, which we demonstrate on phage 7-11.

**Conclusions:**

While MLSA algorithms proved highly useful in computational analysis of transcription regulation, we here established that a simpler procedure is more successful for identifying promoters that are recognized by bacteriophage encoded σ factor/RNA polymerase. We here used this approach for predicting sequence specificity of a novel (bacteriophage encoded) σ factor, and consequently inferring phage 7-11 transcription strategy. Therefore, direct analysis of bacteriophage genome sequences is a plausible first-line approach for efficiently inferring phage transcription strategies, and may provide a wealth of information on transcription initiation by diverse σ factors/RNA polymerases.

## Introduction

Bacteriophages represent a group of viruses that is dominant in the microbial world, which to a large degree outnumber the other life forms in the Biosphere [1,2]. In addition to being dominant over other organisms in terms of their numbers, bacteriophages are also characterized by high population dynamics, with 10^23 ^bacteriophage infections per second.

While bacteriophage genomes are short, analyzing their genomic sequence is noticeably complicated by genetic exchange, which results in genome mosaicism. As a consequence, a large number of genes (almost 80%) in a novel phage does not code for proteins of known functions [1]. On the other hand, bacteriophages share a lot of similar features, like gene expression strategies. For example, genes of a large number of bacteriophages can be designated as the "early", "middle" and "late", based on the temporal pattern of their expression during infection [3].

An additional interest for analyzing bacteriophage gene expression strategies comes with recent occurrence of the bacterial strains resistant to antibiotics, i.e. due to their relevance for bacteriophage-based therapy treatments. Such successful treatments might use the protein products that bacteriophages express during infection, such as the bacterial RNA polymerase inhibitors, cell wall lysins etc. To understand functions that these molecules perform, the analysis of the bacteriophage gene expression strategy during the infection has arisen as an important objective [4,5]. This analysis of the infection strategies commonly heavily relays on experimental measurements; these measurements include the temporal analysis of gene expression by macroarrays, as well as the biochemical analysis of the promoter elements in the genomic sequence [6,7].

The previous strategy is, however, time and resource consuming, which is largely impractical given the exponentially growing pace of sequenced bacteriophage genomes [1,2]. This opens a question of developing more effective methods for acquiring insights into the strategy of bacteriophage infections. An attractive possibility is to extract as much information as possible directly from the bacteriophage genome sequence, through bioinformatic methods - exploring this possibility is the main goal of this paper.

Particularly challenging for the analysis is a large number of bacteriophages that express their own RNA polymerase (RNAP) or σ factor. Some representatives of this group that were recently experimentally analyzed in detail, and which we will include in our analysis here, are Xp10 and phiEco32 [6,7]. At the beginning of the life cycle, these viruses use RNAP of a host bacterium for initiating the gene expression. The following step is the repression of the activity of this holoenzyme, which leads to the shut-off of the expression of bacterial genes. The bacteriophage transcription then switches to using its own RNA polymerase/σ factor, which leads to a completion of the viral gene expression [6-8].

The key element in understanding the transcription strategy of such bacteriophages is the prediction of the promoter elements that these σ factors/RNA polymerases recognize. σ factors and RNA polymerases encoded by sequenced bacteriophage genomes often show resemblance with RNA polymerases and σ factors of other phages and σA group of σ factors. However, the level of homology is almost always insufficient for inferring the specificity of promoter elements that are recognized by σ factors or RNAP that are encoded by a newly sequenced bacteriophage. Consequently, the prediction of the promoter elements reduces to a highly non-trivial bioinformatic task, since it comes to a prediction of a few, possibly ambiguous, ~10 bp motifs in a 50 - 100 kb long sequence. In line with this, it is often reported that promoters with such organization are hardly detectable by bioinformatic methods developed for such problems, which are MLSA (Multiple Local Sequence Alignment) algorithms [9].

As a model bacteriophage to explore inferring gene expression strategy directly from the genome sequence, we will use recently sequenced bacteriophage 7 - 11. The phage has ~90000 bp long double-stranded DNA genome, which infects bacterium *Salmonella enterica *[10]. No experiments are performed on this bacteriophage, so none of its gene expression control mechanisms are known in advance. Our strategy will therefore be to analyze to what extent one can infer a global view of the bacteriophage gene expression strategy directly from its genome sequence.

On the other hand, there is a notable homology of bacteriophage 7 - 11 with phiEco32 phage infecting *E. coli*, which was analyzed in detail before [6]. Consequently, we can use this homology for assessing the obtained bioinformatic predictions. Furthermore, the methods that we will develop on 7 - 11 phage can be directly tested on some of the bacteriophages that express their own σ factor, and for which the complete experimental analysis was done before. With that respect, bacteriophages phiEco32 and Xp10 are of particular interest, since bioinformatic methods alone failed predicting their promoters [6,7].

## Methods

### Bacteriophage 7 - 11 gene prediction and annotation

The genes in bacteriophage 7 - 11 genomic sequence were predicted by GeneMark (version GeneMarkS) [11], with option Phage, and the other parameters set to default values. The obtained predictions are consistent with the GenBank annotation, which was further used in the analyses. To infer functions of the predicted genes BLAST (blastx) was used [12], with parameter options set to default values, and the E-value threshold set to 10^-4^.

### Intergenic region extraction

The upstream intergenic regions were extracted, and further divided in three separate groups, corresponding to *i*) all the genes, *ii*) genes with "+" orientation, and *iii*) genes with "-" orientation. For each group, the orientation of the direct strand corresponds to the gene transcription direction, so that we further search only the direct strand of intergenic regions (note that the promoter motifs are not palindrome symmetric). Having in mind the length of the promoter elements, only intergenic regions longer than 50 bp were extracted. The promoter elements can also overlap with the 3' ends of the upstream genes, so additional 30 bp, corresponding to these 3' ends, were fused to the 5' ends of the intergenic regions; these longer (fused) regions were further used in the promoter searches.

### Detecting phage-specific promoter elements by MLSA algorithms

Method of choice for identifying short motifs that are conserved in a set of DNA sequences are MLSA (Multiple Local Sequence Alignment) algorithms, which were consequently our first choice in the search for phage-specific promoter elements. The Gibbs Motif Sampler and BioProspector - two different implementations of the basic MLSA algorithm, based on Gibbs search - were used in the analysis [13-15].

The Gibbs Motif Sampler was used in the Motif Sampler mode, with the motif length set to 9 bp (for 7 - 11 and phiEco32 intergenic regions) and 18 bp (for Xp10 intergenic regions), and the expected number of motifs per query sequence set to 1. For phage 7 - 11 (and phiEco32) the motif length was set with respect to the approximate length of σA family extended -10 element; note that this element is universally present within σA family, while phiEco32 and 7 - 11 encoded σ elements are distantly related with σA family. For Xp10 the motif length was set with respect to the promoter element of RNAP encoded by the T7 group of phages, which are also distantly related with RNAP encoded by Xp10 [16]. Note that in the last cycle of search, the algorithm adds/subtracts each segment from query sequences based on its impact to the informational content of the alignment [15]. Consequently, the total number of detected motifs per sequence in the final alignment can be also larger or lower than 1. BioProspector was used with the same motif lengths, only the forward strand was searched, and top 3 scores were reported. The other parameters were set to default values in both BioProspector and Gibbs Motif Sampler.

### The phage-specific promoter elements detection through pairwise alignment of the intergenic regions

As the second strategy to identify phage-specific promoter elements, we developed an approach that is based on a pairwise alignment of the bacteriophage intergenic regions; this pairwise alignment was implemented by BLAST (balstn). Our assumption was that this approach is effective in the case when promoter elements are present in a low copy number, in a form of well conserved repeats in the genome. BLAST (balstn) was used with the default options, with an exception that the minimal alignment length was set to 7 bp. Considering the fact that the genes are organized in two divergent clusters (the "+" and "-" clusters), it is reasonable to assume that the first intergenic region in, at least, one cluster should contain at least one motif that matches the phage-specific promoters - otherwise, the genes in the cluster upstream from the promoters would not be transcribed. Consequently, our approach is to pairwise-align the first intergenic region from the "+" cluster (containing all the structural - most probably late genes) both with itself and the remaining intergenic regions. As a back-up option, the pairwise alignment of all "+" intergenic regions is performed - this then accounts for the case that no bacteriophage encoded promoters are contained in the upstream most intergenic region of the structural (late) gene cluster. Finally, a supervised search of "+" intergenic regions is performed, so as to insure that all copies of the motif identified by the pairwise alignment are detected.

### The σA-dependent promoter elements detection

For predicting the promoter elements recognized by host bacterial σ factor, we used a weight matrix search [17,18]. For improving the search accuracy, we used our recent (large scale) alignment of σ70 promoter elements in *E. Coli *[19]. Specifically, this alignment takes into account the following features: i) accurately inferred weight matrices for both -10 and -35 promoter elements, ii) a score which is sampled from the variable distances between these elements, iii) a separate weight matrix which quantifies sequence specificity of the conserved sequences immediately upstream of -10 element ('so called' -15 element).

By using the weight matrices, a specific score can be calculated for any segment of the matching size, based on its nucleotide sequence. The scores are defined so that their maximal value is zero, which corresponds to the consensus -35 and -10 elements, and the optimal (17 bp) spacer [20]. All other scores have negative values, so that better the score, the closer it is to zero. The threshold for the promoter recognition is set manually, as the threshold value is, in principle, provisional [21].

## Results

### The 7-11 genome arrangement

**Figure 1:**
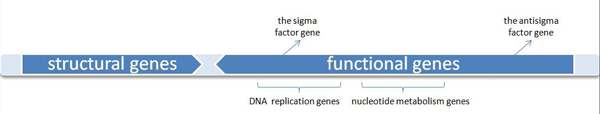
**The organization of the bacteriophage 7 - 11 genome**. Upon cell entry, the genome likely takes a circular form, so that the two gene clusters are divergently transcribed and separated by a long intergenic region which consists of 5' and 3' ends of the genome. The two genes with special importance in transcriptional regulation - σ and anti-sigma factor genes - are marked. The groups of genes involved in DNA replication and nucleotide metabolism are also marked.

The total number of 151 ORF was detected in the phage 7 - 11 genomic sequence, 30 of those oriented in the"+", and the other 121 gene in the "-" transcription direction. The average gene length is several hundred bp, with the largest gene of 2 kb. A significant number of genes do not have homologues in databases with viral or any other organisms' genes so that their potential function cannot be predicted. The genes that possess homologues in databases mostly infer the homology with the phiEco32 bacteriophage. A notable feature of the phage 7 - 11 genome is the sharp grouping of genes into the "+" oriented cluster, which contains the structural and DNA packaging genes, and the "-" oriented cluster, composed of the functional genes (Figure 1); such genome organization is common in bacteriophages encoding their own σ factor/RNAP [6,7]. Note that the genome architecture is likely circular, i.e. 5' and 3' ends of the genome stick with each other, so that there is a long intergenic region separating the two divergently transcribed clusters. In the intergenic region downstream of the two clusters, there are signals for two intrinsic terminators - the signals are located on both the forward and the reverse strand.

The "-" cluster genes (the functional genes) can be divided into two different subgroups - genes involved in processes of genome maintenance and expression (DNA replication and transcription) and genes involved in the metabolism of nucleotides, which are localized in the downstream and the upstream segment of the cluster, respectively. The functional genes also include the σ factor and the anti-sigma factor, whose position is indicated in Figure 1, and which have significant role in transcription regulation. The homology with phiEco32 coliphage indicates that putative function of anti-sigma factor is the host RNAP inhibition, which leads to the shut-off of the host and early phage gene transcription. Once this shut-off happens, further transcription of the phage genes can be exhibited by the bacteriophage-encoded σ factor, which is also indicated in the figure.

### The phage-specific promoter detection in 7 - 11 genomic sequence

Our first approach for the phage-specific promoter detection was using MLSA algorithms (see Methods). These algorithms represent a standard procedure for detection of promoters that are typically ambiguous motifs, which appear in a significant fraction of the intergenic regions. The statistical significance determination of the MLSA algorithms' results is still an open problem [15,22], so the reliability of the predictions is commonly validated based on their robustness - i.e. the predictions obtained by multiple runs of the same MLSA algorithm, or by different implementations of MLSA algorithm (e.g. BioProspector and Gibbs Motif Sampler), should match.

We therefore separately searched for repeated motifs within the "+" oriented, the "-" oriented and all intergenic regions by using BioProspector and Gibbs Motif Sampler. Neither of these searches yielded robust predictions. We assumed that this failure is due to the phage specific promoters appearing in a low copy number in 7 - 11 genome. To account for that, we devised an approach that is well suited for well conserved motifs in a low copy number in 7 - 11 intergenic regions. The approach (see Methods) is based on a pairwise alignment of the intergenic regions within the structural (most likely late) gene cluster, and exploits that the most upstream intergenic region in the cluster is most likely to contain at least one copy of the phage specific promoters. Note that this region often separates the two clusters of divergently transcribed genes (with "+" and "-" orientation) - in an architecture characteristic for the well-known bacteriophage λ - typically has an uncharacteristically large length, and often contains divergent promoters that transcribe both "+" and "-" gene cluster.

By using this approach, we located four putative phage-specific promoters in the upstream-most intergenic region (Table 1). The 12 bp long motif consist of 2 starting bp "TG", followed by the core motif "TGATGT" and an extra "TATA" element. These four ~12 bp repeats are statistically highly significant with estimated E value of ~10^-8 ^in ~4400 bp long sequence (the length of "+" intergenic regions). The core motif from the predicted promoters was further used to track the additional putative promoters that may be missed by the initial alignment due to shorter conserved motif length. Six additional copies were detected upstream of the genes that are indicated in Table 1 which are also statistically significant (at P < 0.05 level). We classified the four copies that we initially identified as the long motifs, and the six additionally detected motifs as the short motifs. This distinction is made based on a significant difference in the conserved motif lengths.

**Table 1:** The putative promoter elements recognized by phage-encoded σ factor in the 7 - 11 genome; The sequences are flanked by the number of the downstream gene (left) and the starting genomic coordinate - 5' end of the "TG" segment for long motifs and of the core "TGATGT"' segment for short motifs (right); The third to eight position in the motifs correspond to the consensus core motif 'TGATGT", while the upstream and the downstream flanking sequences correspond, respectively, to the "TG" segment and the "TATA" element.

Gene	Detected elements	Coordinate
**Long motifs ("late" promoters)**		
1	**tgtgatgttata**	995 bp
1	**tgtgatgttata**	1091 bp
1	**tgt**a**atgttata**	1056 bp
1	g**g**g**gatgttata**	844 bp
	**Short motifs ("middle" promoters)**	
1	a**gtgatgt**gta**a**	1741 bp
1	**t**t**tgatgt**ag**t**c	91 bp
25	a**gtgatgtt**tgg	26138 bp
88	**t**t**tgatgt**atct	56848 bp
116	**t**t**tgatgt**agac	72349 bp
122	cc**tgatgt**a**a**ct	76982 bp

Bases that match the consensus sequence are marked in bold.

We argue that the two groups of the detected motifs (long and short) represent two distinct classes of promoters; this is due not only to the noted differences in the motif sequences, but also due to their position in the genome. Specifically, the long motifs are located upstream of the structural genes, which are likely expressed late in the infection, so that these motifs represent "late" promoters. On the other hand, the short motifs transcribe the genes in the downstream part of the functional gene cluster, which are likely expressed earlier than the structural genes, but later than the genes transcribed by bacterial RNAP; we consequently classify these motifs as "middle" promoters.

Finally, note that the detected motifs are localized downstream with regard to the anti-sigma factor gene: this is consistent with the expectation that transcription by phage-encoded σ factor should be induced, once the transcription by bacterial RNAP is shut-down (which is likely exhibited by the anti-sigma factor).

### The σA-dependent promoter elements detection

We next searched for the bacterial σ^70^-dependent promoter elements, by the improved weight matrix search, as described in the Methods. The choice of the thresholds for the weight matrix search is, in principle, arbitrary, though it is clearly desirable that the promoters are predicted only on "-" strand, i.e. according to the expectation that bacterial σ factor should transcribe only early, functional genes; regarding this, note that bacterial transcription is, most likely, inhibited by the anti-sigma factor later in the infection. Indeed, by searching the intergenic regions, we find that there is a threshold, so that eight promoters are predicted on "-" strand - all of them immediately upstream, or within the functional gene cluster and upstream of anti-sigma factor gene- with no promoters predicted on "+" strand. Such clear positional bias suggests confidence in the obtained predictions. The promoters are shown in Table 2 where one can note a considerable resemblance of the two strongest promoters with the consensus; it is therefore likely that most of the early gene transcription is due to activity of these two strong promoters, which transcribe the functional gene cluster as a long operon. The rest of the promoters likely serve to additionally increase the transcription activity of phage genes - whose activity is typically much stronger compared to those of the bacterial host genes - and also to tune expression of certain genes within the cluster.

**Table 2:** Predicted bacterial σ70-dependent promoter elements in 7 - 11 genomic sequence.

Gene	-35 element ttgaca	Spacer Length	-10 element tataat	Genomic Coordinate (complement)	Motif Score
151	ttgaca	17 bp	tatagt	101:129	-2.78
151	ttgaca	17 bp	taatct	194:222	-2.95
151	ttgcaa	17 bp	taatat	288:316	-3.24
151	ttgccg	17 bp	tagagt	89002:89030	-3.27
96	atgaaa	18 bp	tacaat	59756:59785	-3.67
99	ttgcct	16 bp	tatatt	61590:61617	-3.97
151	ttagta	17 bp	taaaat	323:351	-3.98
99	tttcag	18 bp	tataat	61562:61591	-4.09

Different columns in the table denote (from left to right): i) the downstream gene number, ii) -35 element sequence, iii) the length of the spacer, iv) -10 element sequence, v) coordinate in genome of the promoter element, vi) score of the predicted promoter (see Methods).

### Comparison of the 7 - 11 phage with the phiEco32

Both phiEco32 and 7-11 phages code for their own σ and anti-sigma factors, and there is a highly significant homology between the σ factors of the two phages. Furthermore, there is a high degree of similarity between the genome organization of the two phages, as well as between the layouts and positions of the genes within the clusters. Consequently, our predictions of 7-11 promoters can be further corroborated through comparison with phiEco32, whose transcription regulation has been experimentally analyzed in detail.

**Figure 2:**
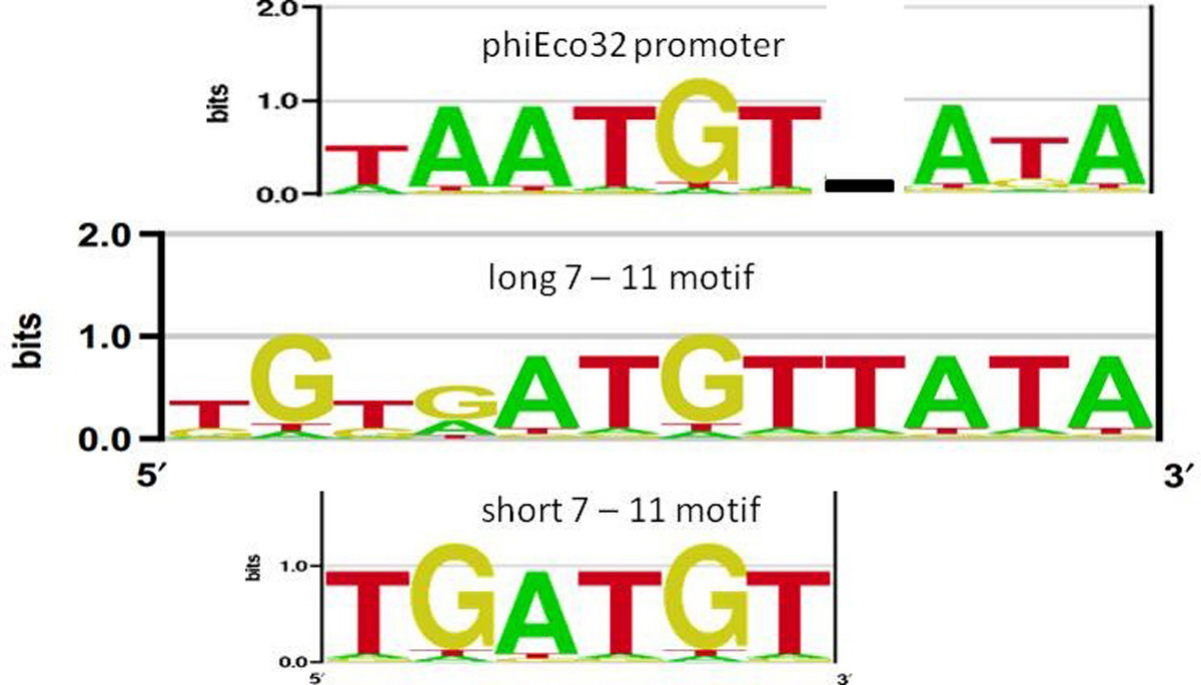
**Comparison of the sequence logos**. The first three lines show the sequence logos for respectively: i) experimentally found phiEco32 promoters [6], ii) 7-11 long motifs (Table 1), iii) 7-11 short motifs (Table 1). The sequence logos were aligned, and one bp gap was introduced in phiEco32 sequence logo, so that similarities between the specificities can be compared. The logos were constructed by enoLOGOS [24].

In Figure 2, we compare the sequence logo of the predicted 7-11 promoters with the experimentally established consensus of the related phiEco32 encoded σ factor. One can see an evident similarity between the two specificities, though the alignment itself is non-trivial due to a one base gap that has to be introduced in phiEco32 consensus. Consequently, the high degree of similarity between the experimentally established phiEco32 specificity, and the detected over-represented 7-11 motifs, provides further argument for their functionality.

**Figure 3:**
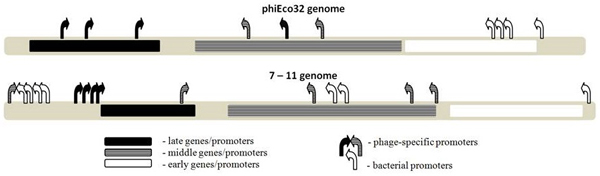
**Comparison of promoter layout and temporal classification for 7-11 and phiEco32**. The upper and the lower line correspond to the promoter layout for phiEco32 and 7-11 genomes, respectively. The color code for the promoter and gene temporal classes is indicated in the figure legend.

Finally, we also note a significant similarity in the promoter layout between the genomes of 7-11 and phiEco32 phages, where the relevant comparison is provided in Figure 3. In both cases, the structural genes are transcribed by the bacteriophage late promoters; the upstream part of the functional gene cluster is transcribed by the bacterial promoters, while the middle bacteriophage promoters transcribe the downstream part of the cluster. Consequently, the equivalent promoter layout further corroborates both the predicted 7-11 promoter specificity, and the classification of the detected promoters to late and middle classes. With that respect, note that the promoter detection was done without using any prior information on the established phiEco32 regulation - i.e. this information was used only to further assess the predictions through homology between the two phages.

### The phage-specific promoter elements detection in the phiEco32 and Xp10 genomic sequences

In both bacteriophages Xp10 and phiEco32, standard bioinformatics methods fail to identify promoters for the bacteriophage-encoded σ factors. We therefore test if the approach that we successfully applied on phage 7-11 can also be used to successfully identify promoters for bacteriophages phiEco32 and Xp10. To that end, we will analyze the two phages (phiEco32 and Xp10) by both MLSA algorithms and the simple method that we developed in searching for 7-11 promoters. We will below first analyze bacteriophage phiEco32 and then Xp10. We will first analyze bacteriophage phiEco32 by both MLSA algorithms and our approach; we will then repeat the same analysis by the pairwise alignment of the intergenic regions (the approach we used for 7-11).

We first separately analyze "+" phiEco32, "-" phiEco32 and total phiEco32 intergenic regions, by BioProspector and Gibbs Motif Sampler; for all three regions the motifs detected by the two implementations of the algorithm differed significantly. The robust predictions were obtained only when the intergenic regions corresponding to all "+" and part of "-" intergenic regions (which correspond to middle temporal class of phage genes, as determined by macroarray measurements) are searched together; the detected motif than matches the experimentally determined consensus "TAATGTATA". Clearly, the MLSA algorithms fail to identify novel phage-specific promoters in the phiEco32 genome, unless relying on the experimentally obtained data. On the other hand, the pairwise alignment of intergenic regions (see Methods) identified 9 bp long motif "tAATGTAtA" upstream from phiEco32 genes: 6, 13, 26, 40, 58 and 68, consistently with experimentally detected phage-specific promoters [6].

We next use MLSA algorithms for searching promoters that are recognized by the single-subunit phage Xp10 encoded RNAP; similarly as with the previously analyzed phages, different MLSA implementations lead to clearly unrelated motifs. In fact, in [7] Xp10 RNAP promoters were identified only after using experimental information on temporal gene expression. On the other hand, the pairwise alignment of the intergenic regions readily identifies two perfect 43 bg long repeats within the long intergenic region upstream of the structural gene cluster, which are statistically highly significant. Note that it is common among bacteriophages to have few copies of highly conserved and strong bacteriophage encoded promoter elements, which transcribe a large fraction of bacteriophage genes as a long operon. This is also the case for bacteriophage Xp10, where the two long repeats contain two ~20 bp experimentally confirmed promoter elements. Consequently, the pairwise local alignment of the intergenic regions can be used for accurately identifying the phage promoters in two phages (phiEco32 and Xp10), where more standard methods (MLSA algorithms) faced considerable difficulty.

## Discussion

Identifying promoters in phage genomes is a key step in analyzing temporal regulation of bacteriophage transcription. Together with more straightforward predictions of genes and their function, it allows efficiently inferring bacteriophage infection strategies. As an illustration, we will below discuss predicting 7-11 gene expression strategy; note that this strategy is inferred by bioinformatic methods alone, directly from 7-11 genome sequence.

Based on the promoter layout in the genome, predicted gene function, and gene clustering, 7-11 life cycle can be summarized as follows: Upon virus entry in the cell, host RNAP starts to transcribe bacteriophage "early" genes from the bacterial σ^70^-dependent promoters. The early genes include the upstream part of the functional gene cluster, together with the anti-sigma factor gene. Since bacterial promoters are located upstream of the "early" genes, these genes are likely transcribed in a form of a long operon - this feature is also detected in a number of other bacteriophages [23].

When the anti-sigma factor is expressed, it abolishes bacterial RNAP activity, so that transcription of both bacterial and early bacteriophage genes is inhibited. The expression of the remaining genes (middle and late) is then directed by the promoters recognized by the phage-encoded σ factors. Interestingly, these promoters are located upstream of the genes coding for the phage σ factor, so that it is transcribed by both phage-encoded and bacterial promoters. This indicates that the initial amounts of the phage σ factor - which are necessary to commence the gene expression to the phage-specific promoters - are transcribed from the promoters recognized by bacterial σ factor. When these promoters are inhibited by the anti-sigma factor, the σ factor expression continues from the phage-specific promoters. Consequently, the σ factor activity remains high both in the beginning and later in the infection. Genes with this transcription pattern are classified as middle genes, they are located in the downstream part of the functional gene cluster, and transcribed by the predicted middle promoters (short motifs). More specifically, this temporal class often includes genes that are involved in the disruption of host transcription or translation mechanisms. Consequently, for phages of pathogenic bacteria, inferring the transcription strategy may allow pointing to the genes/proteins with potentially therapeutic applications.

Finally, only the predicted promoters that are localized upstream of the phage structural and DNA packaging genes - which are organized in "+" cluster - have the extra "TATA" element. We propose that this extra element is responsible for the late transcription, possibly by providing a sufficient promoter strength late in the infection. Transcription of the late genes produces phage structural proteins, and completes the bacteriophage life cycle, so that a large number of virions can enter the new infection cycle.

## Conclusions

We here started from the recently sequenced genome of bacteriophage 7-11. The main goal was investigating if the main determinants of phage transcription regulation (promoters), and consequently the phage infection strategy, can be inferred directly from the phage genome sequence. We found that widely used MLSA algorithms, which proved highly useful in computationally analyzing transcription regulation in general - are not well suited for the task of detecting bacteriophage promoters. On the other hand, a more simple approach based on a pairwise alignment of a subset of phage intergenic regions, was here shown to lead to a robust prediction of the phage promoters. This approach is straightforward to implement, as it is based on well-established methods for pairwise sequence alignment (e.g. BLAST).

We here predicted the sequence specificity for novel, phage 7-11 encoded, σ factor. Importantly, for this prediction, we located few ~10 bps motifs in ~100 kbps of the genome sequence; locating the motifs was based only on the genome sequence. The predictions were afterwards further corroborated based on similarity with bacteriophage 7-11, as no experimental information on 7-11 transcription regulation is available. More generally, bacteriophages code a wealth of different σ factors and RNAPs. The results presented here provide confidence that sequence specificities for a number of those important enzymes can be inferred in a highly efficient manner; this may in turn significantly contribute to the understanding of transcription initiation that provides a major checkpoint in gene expression regulation.

In summary, we argue that the analysis presented here provides a highly efficient, first line approach, for analyzing bacteriophage infection strategies. Historically, analyses of bacteriophages lead to a number of major paradigms in gene expression regulation and may find important medical applications in the future. The methods for more efficient analysis of bacteriophage infection strategies - such as these considered here - may help accelerating the pace of these discoveries.

## Competing interests

The authors declare that they have no competing interests.

## Authors' contributions

MD conceived the work. JG and MD performed the analysis, interpreted the results and wrote the paper.

## Declaration

Publication of this article has been funded by the Swiss National Science Foundation under SCOPES project number IZ73Z0_152297.

This article has been published as part of *BMC Evolutionary Biology *Volume 15 Supplement 1, 2015: Selected articles from the IX International Conference on the Bioinformatics of Genome Regulation and Structure\Systems Biology (BGRS\SB-2014): Evolutionary Biology. The full contents of the supplement are available online at http://www.biomedcentral.com/bmcevolbiol/supplements/15/S1.
